# Sanitation of Meat Markets*Read at Corpus Christi meeting of the Association of Texas Health Officers.

**Published:** 1908-07

**Authors:** B. T. Young

**Affiliations:** Assistant State Health Officer, Austin


### Sanitation of Meat Markets.*

BY DR. B. T. YOUNG, ASSISTANT STATE HEALTH OFFICER, AUSTIN.

In the work of a health officer there is no more important feature than the inspection and proper regulation of the country slaughter "pen." I say "pen," for it is more often a pen than a house, and usually a hog pen at that. Many so-called slaughter houses consist of a plank fence enclosing a part of a ditch, a number of hogs and three poles put up "wigwam" fashion in the center where the animal is cleaned, some one keeping the hogs and buzzards away until this "cleaning" process is over.

No other institution concerned in supplying food to the public has been allowed to exist with so little regard to the laws of sanitation or rules of common cleanliness, and this, too, in handling one of the most easily contaminated of all food products. It is hard to conceive of a place so vile and filthy, much less can we think of men who will supply food to the public from such places.

Nearly all butchers are to blame for the existing conditions, though a few of them have endeavored so far as they knew to build and maintain sanitary slaughter houses. They have gone so far as to build houses, put in cement floors and removed the offal a short distance, but they are prone to neglect the important feature of scrubbing and washing after each killing. The places soon become foul-smelling and filthy from bits of blood, offal and washings that are allowed to dry on the floors, walls and tools. The

*Read at Corpus Christi meeting of the Association of Texas Health Officers.

cleaning process is too often left to half-grown negroes, whose idea of cleanliness is to appear clean on the outside. As one negro killer said on being asked about some muddy water, "No, suh; we don't use dat on de meat; dat's whar I washes mah feet afer I gits through." Do you wash your feet before going into this work? "No, sah; jes' whin I git 'em dirty in dere." The trouble with them is that they don't know how to be clean. They need education, and it is the health officer's duty in every case to instruct them.

The average slaughter house, where the poles have been supplanted by such, consists of a mere shell of a house built of rough, undressed lumber, inviting everything that touches it to stick; plank floors; open walls; holes for windows and doors, never screened—a harbor for rats and mice, and a constant source of flies. It is so constructed that the offal can be rolled out of the building through a hole in the wall or dropped through a trap in the floor. The hogs are depended upon to dispose of the offal, which they do more or less thoroughly as their number is great or few. If there are not enough hogs, there will be a residue of offal left to putrify, attract flies and raise a stench. The water waste always furnishes an excellent wallow under the house. This wallow is full of hides, hoofs, scrapings of hogs, scraps of offal, manure, etc. Putrid gases from this place rise up through the building to poison the freshly dressed meat and contaminate the whole place and air for several hundred feet around, and the fresh meat coming from these places often has the odor of these wallows.

Millions of flies infect these places—house flies and blow flies. They feed upon the filth outside, smear it over themselves, and swarm back into the slaughter house, track across the floors, tables, tools, and even the meat, inoculating everything they touch with the infection from scraps of carcasses outside. It is impossible for meat to be dressed in such a place without becoming contaminated, and it is marvelous that there is no more diseases directly traceable to these slaughter houses than there is.

A model slaughter house should be situated in a place sufficiently secluded and isolated so as not to be unduly dusty, and not to create a nuisance; have a cement floor, built on an incline toward the center, where there should be a trough which would convey the offal. blood and washings outside the building into the offal tank. The margins of the floor should be raised six or eight inches so as to make a perfectly water-tight killing basin, in which any amount of water may be used without leaving a residue in cracks and crevices to putrify and furnish such admirable culture media for the development of germs. The building should be tightly constructed with plenty of windows and door, for light and ventilation; these windows and doors to be well screened with 14-mesh wire gauze; the inside walls should be lined for at least six feet from the floor with some washable material, such as galvanized iron, tin or oilcloth. Adequate lavatories should be provided; and soap, water and clean towels be provided at all times, and employes be required to make use of same. No toilet room in any slaughter house should communicate directly with any room where animals are killed, or where meat is handled or kept. The hide room should be separate and distinct from the killing room. A slaughter house should always have some means of getting water under pressure for the purpose of properly washing the meat and washing walls and floors at least once a day. In a town where there is a public water supply they should always have a hydrant, but, in case this convenience is not to be had, a tank placed on top of the slaughter house and kept full would suffice; this water, however, should never come from a well within 100 feet of the killing yard, nor from below the place at any distance. The offal tank should be constructed of galvanized iron, zinc or a zinc-lined wooden box, and placed upon wheels. It should be large enough to hold the offal, blood and washing from at least one killing and emptied immediately after the slaughter house is cleaned. It should be put into a pot and boiled and then may be emptied into the hog pen, which should be at least 100 feet from the slaughter house, preferably 100 yards.

The feeding of the material to the hogs is in a measure objectionable, as there is some danger of trichina; but as the feeding of this is quite an item to the butcher, and as it is practicably impossible to burn it, I think it all right, provided the material be well cooked, and the hogs be taken out of this pen and grain fed for at least six weeks before killing.

In conclusion, I will state that the butcher's wagon deserves scrutiny also. However well regulated the slaughter house, if the wagon is filthy, greasy, swarming with flies, and teeming with bacteria, the meat will be contaminated. A butcher's wagon, and every vehicle used to transport fresh meat, should never be allowed to become veneered over with organic matter. Frequent applications of concentrated lye and liberal treatment with the hose will keep the wagon clean. By rigid observance of the laws of cleanliness all along the line—at the slaughter house, in the wagon, in the butcher shops and meat markets, in the kitchen—we shall save many a home from sorrow and save many a good citizen for our State.

